# Longitudinal follow-up of oxidative stress and DNA damage parameters in detergent workers

**DOI:** 10.4103/0019-5278.50724

**Published:** 2009-04

**Authors:** Masoud Mashhadi Akbar Boojar, Faranak Goodarzi

**Affiliations:** Department of Biology, University of Tarbiat Moalem, No. 49, Dr. Mofateh Avenue, Tehran, P.O. Box 15614, Iran

**Keywords:** Antioxidant enzymes, detergent exposure, DNA damage, environment monitoring, lipid peroxidation

## Abstract

**Background:**

The aim of this study was the follow-up of work place enzyme and detergent dust exposure effects and smoking habit on DNA damage parameters of workers and the evaluation of their antioxidant enzyme activities and lipid peroxidation with regard to bag-filter installation in the work place.

**Material and Methods:**

All investigated parameters were studied in a group of 153 workers of enzyme-free detergent production plant (E-free) and a group of 138 workers of enzyme-plus detergent plant (E-plus) and compared with 45 controls 7.2 years before and 3.1 years after filter system installation. The following methods were used: antioxidant enzymes by an ultraviolet–visibles spectrophotometer, malondialdehyde (MDA), 8-hydroxy-2'deoxyguanosine (8OH-2'dG) by high-performance liquid chromatography, trace elements by atomic absorption spectroscopy, and comet assay by single cell gel electrophoresis.

**Results:**

Compared with controls, significant increases were observed in both detergent-exposed groups with respect to the levels of MDA, antioxidant enzyme activities, and DNA damage parameters, including 8OH-2'dG, endonuclease III-sensitive sites, and DNA strand breaks, with enhancement effect of smoking before filter system installation. After filter installation, besides significant decrease in the detergent and enzyme dust of airborne and oxidative stress indicators, there was improvement in all DNA damage investigated parameters at the end of this study. The levels of cumulative exposure index of detergent dusts decreased significantly after airborne improvement and showed positive correlation with internal biochemical parameters.

**Conclusions:**

We concluded that high levels of enzyme and detergent contents of work place dusts had a cumulative effect and smoking had a synergistic effect on the imbalance of antioxidant status and lipid peroxidation, suggesting that oxidation stress is important in the occurrence and progression of DNA damage over this study. Detergent and enzyme contents in respirable and total dust had the main role and sufficient potential in their genotoxicity.

## INTRODUCTION

Free radicals are generally very reactive molecules, produced continuously during the course of normal oxidative metabolism and generated by many exogenous agents. The most important reactions of free radicals in aerobic cells involved molecular oxygen, its radical derivatives (superoxide anion and hydroxyl radicals), and peroxides.[1] Reactive oxygen species (ROS) cause damage to cells by reacting with molecules such as lipids, proteins, and DNA.[2] Oxidative DNA damage can produce base and sugar lesions, strand breaks, and base-free sites,[3] which, if left unrepaired, plays an important role in a number of disease processes.[4] Urinary 8-hydroxy-2'-deoxyguanosine (8OH-2'dG), one of the oxidative-modified DNA bases, is a typical biomarker of oxidative stress and plays some role in carcinogenesis.[5] In addition, recent advances in the single cell gel electrophoresis (SCGE) allow the measurement of *in situ* DNA damage in lymphocytes or mononuclear cells.[6]

For many years, occupational exposure to exogenous genotoxic chemical agents has been considered as a particular domain for DNA damage study. In this domain, workers of detergent industries can be exposed to aeroallergens, including detergents and additive enzymes, during production processes that are not present in the general environment.[7] Many histological studies revealed that eosinophils, neutrophils, and phagocyte cell types infiltrate into the lung consequent to the exposure to high levels of detergent and enzyme aerosols.[8] These processes tend to enhance the stimulation of ROS production and then several indices of oxidative damage are enhanced on target biomolecules, including DNA.[9] The amount of damage depends on the rate and the extent of ROS production and the capacity of enzymatic protective mechanisms. The superoxide radical is dismutated by superoxide dismutase (SOD) to hydrogen peroxide (H_2_ O_2_) and molecular oxygen.[10] Hydrogen peroxide, as an oxidizing agent, is converted to oxygen and water by catalase (CAT) and glutathione peroxidase (GPX). GPX protects the membrane lipids from oxidative damage and detoxifies the organic peroxides. It can also act on organic hydroperoxides.[11] In recent years, a few studies have shown the relation between lipid peroxide, enzymatic antioxidants, and exposure to aeroallergens,[12,13] although the antioxidant indices levels and oxidative DNA damage among detergent-exposed workers and their follow-up have never been reported. In the present study, two groups of workers were longitudinally exposed to enzyme and detergent dusts of work place in two different detergent production plants. Accordingly, the aim of this study was to investigate the effects of work place enzyme and detergent dust, smoking habit, and improvement of inhalation air of work-place on the levels of workers' lipid peroxidation, antioxidant enzyme activities, and the status of DNA damage parameters in different monitoring times.

## MATERIALS AND METHODS

### Study population and plant description

The primary target population consisted of 334 male workers who were employed on a permanent basis in a detergent industry, of whom 291 workers were retained as target group after application of exclusion criteria and consideration of workers who left employment or moved and/or canceled their participation through this study. Of these, 153 workers were in the enzyme-free detergent production plant and 138 in the enzyme-plus detergent production plant, where total production volume of the two kinds of detergent powders were equal over this study. In addition, protease enzyme was used in detergent production. All workers were exposed to dust of the work place during manufacturing processes, mixing, filling, and packing on the basis of 7–8 h/day and 5-day-week. They were interviewed periodically with a medical detailed by internists and underwent periodical laboratory examinations once a year.

The control group consisted of 45 workers without environmental and work place exposure to detergents and enzymes with the same pre-conditions and time frame. They were screened through an initial questionnaire with regard to the same occupational physician, similar socioeconomic status (salary, education), and similar workload characteristics. They were free of exposure to heavy metals, organic solvents, and aeroallergens. They had clinical examinations once a year and worked in the same geographical region and climatic conditions. All participants in this study had similar diets, which were served in a 6-day cycle menu in their work place. Smokers included all current smokers, smoking on average 12 cigarettes daily and were allowed to smoke during their work shift. Before this study, they had 3.5 ± 0.6 years of smoking. Non-smokers had not smoked at least one cigarette a day for as long as 2 years. The participants of both the groups were interviewed by a questionnaire for medical and occupational history, amounts of tobacco smoking, daily consumption of non-alcoholic beverages, dietary habits (especially fruits, vegetables, fish, milk products), and for drug use. Subjects were excluded if they had any history of occupation in the detergent or enzyme industry, if they adhered to any dietary restrictions, or if they were exposed to industrial dust and other harmful agents to the nervous, immune, and respiratory systems. The participants did not take anti-inflammatory agents, steroid hormones, antioxidants, or vitamin supplements. They were also asked to refrain from any form of exercise during the medical and laboratory survey period. They had no overt clinical cardiovascular disorders, neuromuscular dysfunction, or respiratory disease before employment in the detergent industry. Cases with diabetes mellitus and rheumatoid arthritis were excluded. According to pre-employment clinical records, all participants in this study were in good health at the first survey. Each participant underwent a complete examination by the same certified specialist with a special experience in the diagnosis of certain abnormalities and was unaware of participant's occupational conditions over this study.

### Questionnaire

The questionnaire included demographic data, current smoking habit, and work-related symptoms.

### Study design

This study was conducted from November 1991 to December 2002 for exposed workers and controls in three different times of monitoring, including: first monitoring, at the time of employment; second monitoring, 7.2 ± 0.3 years after employment (just before installation of bag filters in the work place); third monitoring, 3.1 ± 0.2 years after second monitoring.

### Selenium in plasma

Recommended method for determining selenium in plasma specimens was flameless atomic absorption spectrometry (AAS) (Unicam; model 929. Cambridg, England) with deuterium background corrector.[14]

### Vitamin C and iron in plasma

Collected heparinized blood was centrifuged to obtained plasma. Ascorbic acid in plasma was oxidized by Cu^2+^ (from copper sulfate solution) to form dehydroascorbic acid, which reacts with acidic 2,4-dinitrophenylhydrazine to form a red bis-hydrazon, which is measured photometrically at A520 (normal: 0.4–1.5 mg/Liter).[15] Diluted plasma (1:1 with deionized water) was analyzed for iron by atomic absorption spectroscopy (Unicam; model 929). The method was as described by the “International committee for standardization in hematology.”[16]

### Antioxidant enzyme assay

Heparinized whole blood was centrifuged for 10 min at 2000 g and then aspirated off the plasma. The erythrocytes were then washed four times with 3 ml of 0.9% sodium chloride solution. Aliquots of the washed erythrocytes were lysed.

The SOD activity in erythrocytes was measured according to the method of Misra and Fridovich,[17] in which the activity in erythrocytes was on the basis of their ability to inhibit free radical chain oxidation in which O_2_^−^ was a chain-propagating radical and the autooxidation of epinephrine was included. Human erythrocyte SOD (Sigma, Lenexa, KS, USA) was used as a standard and the activity was expressed in U/g. Hb. CAT activity of the erythrocytes after washing thrice with isotonic sodium chloride was measured at 25°C as previously described.[18] 1 k of CAT activity was defined as the rate constant of the first order reaction. GPX activity in whole blood was measured by the method of Paglia *et al*.[19] Heparinized whole blood (0.05 ml) was diluted with 1 ml diluting agent, incubated for 5 min, and then 1 ml of double strength Drabkin's reagent was added and mixed well. GPX catalyses the oxidation of glutathione (GSH) by cumene hydroperoxide. In the presence of GSH reductase and nicotinamide adenine dinucleotide phosphate (NADPH), the oxidized GSH is immediately converted to the reduced form, with the concomitant oxidation of NADPH to NADP^+^. The decrease in absorbance at 340 nm was measured by a spectrophotometer.

All analyses were performed on the spectrophotometer – UV Gilford (model 250, Nova Blotech, San Diego California). Activities were expressed as units per gram of hemoglobin (Hb).

### Hemoglobin

The hemoglobin in the erythrocyte lysates was estimated by the method of Drabkin and Austin.[20]

### Malondialdehyde (MDA) in plasma

The MDA concentration in plasma was measured by the high-performance liquid chromatography technique (model: crystal 200; Beckman Instrument Inc., Fullerton California) in which the MDA–thiobarbituric acid (TBA) adduct was separated.[21] Briefly, plasma lipoperoxides were hydrolyzed by boiling in diluted phosphoric acid. MDA was reacted with TBA to yield the MDA–TBA adduct. The protein-free extract was fractionated on a C_18_ column of octadecyl silicagel to separate the MDA–TBA adduct by elution with methanol/phosphate buffer and quantified by a spectrophotometer at 532 nm (model 4225; Unicam, LCD/Analytical).

### Determination of 8OH-2'dG in urine

8OH-2'dG levels in the urine were measured essentially as described previously.[22] Briefly, an automated column switching method for 8OH-2'dG is based on the unique selectivity of the integral porous carbon column for purines. Samples were injected onto a C8 column and the band containing 8OH-2'dG was then quantitatively trapped on a carbon column. The selectivity of the carbon column for 8OH-2'dG allows elimination of interfering peaks by washing the column with a second mobile phase and then eluting 8OH-2'dG to an analytical C18 column with an identical mobile phase containing adenosine to displace 8OH-2'dG. Detection with series colorimetric electrodes provides qualitative certainty for 8OH-2'dG peak by response ratios.

### Measurement of DNA base oxidative damage

1. Blood sampling: A 2 ml blood sample was mixed with an equal volume of phosphate-buffered saline (PBS). Three milliliters of Histopaque-1119 (Sigma) was layered in 10 ml polypropylene conical tubes. The diluted blood was carefully layered over the gradient and then spun at 300g for 15 min at 4°C. The opaque layer containing peripheral blood mononuclear cells (PBMCs) was aspirated and transferred to separated siliconized glass tubes and washed with 5 ml PBS. The cell pellet was resuspended with PBS to give a final cell concentration of 6 X 10^4^ cells/ml.
2. The principle of the SCGE (or comet assay) for the detection of DNA strand breaks has been described previously.[6] Briefly, microscopic slides were coated with melted 1% standard agarose and dried down on a warm heater. PBMC suspension (30*µ*l) was mixed with 60 *µ*l low melting point agarose (LMA) and added to the slides. The slides were covered with a coverslip and kept in a refrigerator for 5 min. Then, the coverslip was removed and the slides were immersed in a jar containing lysing solution (2.5 M NaCl, 0.1 M EDTA, 10 mM Tris, and 1% Triton X-100) at 4°C for 1 h. After lysis, the slides were gently placed in an electrophoresis tank in 0.3 M NaOH and 1 mM Na_2_ EDTA for 40 min. Electrophoresis was then carried out at 25 V and 300 mA for 30 min. After electrophoresis, the slides were neutralized in 0.4 M Tris buffer (pH 7.5), rinsed in distilled water, and stained with approximately 50 µl of SYBR Green 1. After this, each slide was immediately viewed by fluorescence microscopy. Microscopic slides were coated with the mixture of PBMC and LMA as described above. After cell lysis, the slides were washed three times in a buffer containing 40 mM HEPES, 0.1 KCl, 0.5 mM EDTA, and 0.2 mg/ml bovine serum albumin (pH 8.0). Then, 50 *µ*l of either endonuclease III (1 mg/ml) or formamidopyrimidine glycosylate (22 pg/*µ*l) was placed on to agarose (a total of two separate slides) and the gel was covered with a coverslip and incubated at 37° C for 45 min. Comet in each gel was analyzed using a charge-coupled device camera and komet 3.0 image analysis program (Kinetic Imaging Ltd., Liverpool, UK). The frequency of DNA single strand breaks was determined by measuring the percent of DNA in the tail portion (tail DNA) from a total of 50 mononuclear cells per sample (25 cells from each of the two replicate slides). The data were further standardized using an internal standard, obtained from the K562 human erythroleukemia cell line, as described previously.[23] The amount of oxidized pyrimidines (endonuclease III-sensitive sites) or purines (formamidopyrimidine glycosylase [FPG]-sensitive sites) in DNA was measured by subtracting the tail DNA without enzyme treatments from the tail DNA with enzyme incubation.[24] Observation and analysis of the results were carried out by the same experienced person and were conducted in a blind way, i.e. the observer had no knowledge of the identity of the slide.

### Detergent monitoring and enzyme analysis in the air of the work place

The sampling strategy included static measurement for total detergent dusts and parallel stationary environmental monitoring of respirable (below 0.8 *µ*m) and non-respirable dust, in which the samples were obtained using high-volume samplers and high-efficiency glass fibers. The Galley samplers were strategically positioned at fixed locations in the plant, which were best suited to estimate exposure conditions of the employees. Samples were taken for 10 days and each day for 8 h at a flow rate of 36 cfm. Total particulate concentrations were determined gravimetrically and reported as micrograms of total dust per cubic meter of air (*µ*g/m^3^).[25]

The size–mass distributions of airborne dust particles were determined by use of a 1 cfm eight-stage cascade impactor. The impactor was positioned adjacent to the high-volume samplers. Eight-hour time-weighted average personal breathing zone exposures to total particulates was determined using standard sampling techniques. Particulates were collected at a flow rate of 0.07 cfm on 37-mm diameter, 0.8 *µ*m pore size, polyvinyl chloride membrane filters that were contained in three-piece closed face cassettes. Fraction particle size was on the basis of filter pore size used in particle entrapment and passage. Respiratory detergent dust was considered as non-respirable fraction plus respirable mass.

The estimation of proteolytic enzyme content in a sample was based on the reaction of the enzyme with N, N-dimethyl casein. Amino acids formed by reaction with the enzyme were caused to react with 2,4,6-trinitrobensulphonic acid to form a colored complex. Enzyme concentrations were reported as micrograms of proteolytic activity per cubic meter of air (*µ*g/m^3^) on the basis of a pre-determined assay that Esperase enzyme contained 2.6% pure crystalline-proteolytic enzyme.[25] The recommended level of exposure to proteolytic enzyme was calculated from the total enzyme dust threshold limit value (TLV) of 0.06 *µ*g/m^3^ proposed by the American Conference of Governmental Industrial Hygienists (ACGIH).[26] The calculated enzyme-equivalent expression for proteolytic enzyme was 3.9 *µ*g/m^3^.[27]

### Cumulative exposure index (CEI)

This parameter was calculated for each subject by multiplying the average annual airborne detergent or enzyme concentration in respirable dust characteristic of each job by the number of years in which this activity was performed. The average daily intake of detergent from air was estimated on the assumption that there was 100% absorption of respirable detergent dusts from 6.66 m^3^ daily inhalation volume (20 m^3^ daily inhalation volume for a 70 kg adult] × 1/3 [rate of daily work shift time] = 6.66 m^3^) for a complete period of daily work shift. The index was expressed in micrograms of particulate per cubic meter of air times duration of exposure in years.

### Detergent measurement in water

The detergent analysis was on the basis of the methylene blue reaction as the precise and useful method for overestimating of anionic surfactant content of water, named as methylene blue active substances reaction. At first, the surfactant was separated by sublation process from the water sample, which yields a dried residue relatively free of non-surfactant substances. Then, the extracted material reacted with methylene blue solution. The process was followed by an aqueous backwash and measurement of the blue color in the CHCl_3_ by spectrophotometry at 652 nm.[28] The detection limit was equal to or greater than 10 *µ*g/L. When obtaining samples for chemical analysis, great care was taken against contaminating the water samples. Sampling bottles were carried in suitable crates and delivered to the laboratory without delay.

### Water mineral analysis

The concentration of manganese,[29] lead,[30] zinc,[30] and iron[31] in the water was determined by atomic absorption spectrometry.

### Nitrate and hardness of water

The method used for the measurement of nitrate was based on photometric analysis after reduction to nitrite.[32] The hardness of water samples was expressed as calcium carbonate concentration per liter. It is usually calcium hardness that predominates.[33]

### Statistical analysis

The χ^2^ and z tests were used for testing differences in the mean values of enzymatic and non-enzymatic parameters of plasma and erythrocytes, concentrations of dust–enzyme and total dust of work place air, and mean values of water minerals. The z test was also used for testing differences in comet assays results. Group comparison was assessed by ANOVA. A level of *P* < 0.05 was considered statistically significant. In the testing for relationships, the entire involved populations and parameters in second and third monitoring were considered separately and the Pearson's correlation was calculated.

## RESULTS

Table 1 demonstrates the selected demographic characteristics and smoking habits of the participants. The mean age of the study workers was around 27 years for E-plus and about 25 years for E-free detergent-exposed workers. There was no significant difference between each group of detergent producer workers and controls in terms of age, height, total time in industry, weight, and smoking status. Although the percentage of smokers in both detergent-exposed workers were higher than controls, the difference was not significant.

**Table 1:** General characteristics of the study groups

Characteristic	Detergent-exposed workers		Controls (*n* = 45)
		
	Enzyme-plus detergent (*n* = 138)	Enzyme-free detergent (*n* = 153)	
Age (Yrs)	27.4 ± 3.6	25.2 ± 4.2	27.0 ± 5.1
Height (cm)	173.6 ± 3.3	177.3 ± 5.1	174.6 ± 4.4
Weight (kg)	77.4 ± 7.4	76.2 ± 8.1	74.2 ± 6.7
Current smoking ([12 ± 5] cig/day)	57 (41%)	58 (38%)	16 (35%)
Non-smoking	81 (59%)	95 (62%)	29 (65%)
Water (glass/day)	10.2 ± 3.6	9.4 ± 3.1	8.2 ± 3.8
Tea (cups/day)	3.4 ± 1.3	2.3 ± 1.8	2.6 ± 1.4

#Values are mean ± SD and a level of *P* < 0.05 was considered significant.

The distribution of stationary sampling dust levels and their enzyme contents are provided in Table 2. At first monitoring, total dusts in the enzyme-plus detergent plant ranged from 930 to 1580 *µ*g/m^3^, with a mean value of 1346 *µ*g/m^3^, and in the enzyme-free detergent plant these ranged from 1065 to 1750 *µ*g/m^3^, with a mean value of 1428 *µ*g/m^3^. Both mean values were greater than the particulate level guideline (1000 *µ*g/m^3^) used by the soap and detergent industry association.[34] Respirable fraction of total mass of inhaled particles were around 40–45% in most breathing zones. Enzyme levels in total dust were around 0.5%, of which about 60% were in the respirable portion. The mean concentrations of enzyme were greater than the TLV of 0.06 *µ*g/m^3^ proposed by the ACGIH.[26] The concentrations of airborne parameters did not change significantly before bag-filter installation but decreased significantly after improvement of work place and at the end of this study. In new conditions of the work place, total dust and the level of enzymes were considerably lower than the Soap and Detergent Industry Association (SDIA) and TLV levels, respectively. In addition, the filtration lowered the respirable portion to 37% in the field of enzyme plus detergent plant and the levels of airborne parameters did not differ significantly.

**Table 2:** The work place air pollution concentrations and cumulative exposure index in different monitoring times in the field of detergent production

Parameter	First monitoring	Second monitoring		Third monitoring
			
		Before bag filter	After bag filter	
(+)E total dust (*μ*g/m^3^)	1346 ± 214#	1495 ± 246	359 ± 54†	404 ± 64†
(—)E total dust (*μ*g/m^3^)	1428 ± 259	1577 ± 226	393 ± 58†	442 ± 54†
Enzyme in total dust (*μ*g/m^3^)	6.57 ± 2.6	7.33 ± 2.16	1.88 ± 0.28†	2.10 ± 0.34†
(+)E respirable dust (*μ*g/m^3^)	574 ± 93.5	659 ± 124.7	135 ± 19.3†	152 ± 24.3†
(—)E respirable dust (*μ*g/m^3^)	583 ± 184	618 ± 102	156 ± 25.2†	176 ± 23.6†
Respirable enzyme (*μ*g/m^3^)	3.77 ± 1.43	4.28 ± 1.31	1.15 ± 0.18†	1.28 ± 0.19†
Respirable enzyme CEI (*μ*g/m^3^)	783 ± 244	912 ± 262	232 ± 37†	262 ± 40†
Respirable detergent CEI (*μ*g/m3)	123 ± 21	140 ± 26	28 ± 4†	32 ± 5†

(+)E, field of enzyme plus detergent; (—)E, field of enzyme-free detergent.

# Values are mean ± SD and a level of *P* < 0.05 was considered significant.

† Parameters that their mean values differ significantly from values of first monitoring.

The chemical composition of consumed water at all stages of monitoring is presented in Table 3. The mean concentrations of all minerals, nitrate, and hardness did not exceed the WHO guideline values (Pb = 50 *µ*g/L, Mn = 50 *µ*g/L, nitrate = 10 mg/L, Zn = 500 *µ*g/L, Fe = 300 *µ*g/L) and changed insignificantly over this study. In addition, the levels of detergent in drinking water were below the detection limit of 0.01 mg/L.

**Table 3:** Chemical characteristics of water in different monitoring times

Parameter	First monitoring	Second monitoring	Third monitoring
Pb (*μ*g/L)	6.78 ± 2.06	7.44 ± 2.12	5.86 ± 1.84
Zn (*μ*g/L)	264 ± 44	306 ± 52	238 ± 41
Mn (*μ*g/L)	12.5 ± 2.7	16.3 ± 3.5	18.6 ± 4.2
Fe (*μ*g/L)	197 ± 46	228 ± 38	173 ± 43
Nitrate (mg/L)	3.44 ± 1.06	4.26 ± 0.92	4.76 ± 1.14
Hardness (mg/L CaCo_3_)	108 ± 26	116 ± 32	88 ± 23
Anionic surfactant (*μ*g/L)	< 10	< 10	< 10

#Values are mean ± SD and a level of *P* <0.05 was considered significant.

Mean values of all parameters do not differ significantly from the values of controls.

The mean values of biological parameters in the subgroups of detergent-exposed workers and controls are illustrated in Table 4. Mean activities of antioxidant enzymes (SOD, CAT, and GPX) and mean concentration of MDA were significantly higher in the second and third monitoring in both detergents-exposed workers as compared with the same group at first monitoring and controls. Recent parameters were significantly higher in current smokers than in non-smokers in enzyme-plus and enzyme-free detergent-exposed workers at second and third monitoring, except GPX. Current smokers revealed insignificantly higher levels of GPX activity than non-smokers in second and third monitoring. At first monitoring, in spite of insignificant variation in the parameters among controls and two detergent-exposed groups, they did not differ significantly between their subgroups. In third monitoring, the mean values of MDA, SOD, CAT, and GPX decreased significantly in all detergent-exposed workers as compared with second monitoring. In addition, current smokers revealed higher levels in these indicators as compared with non-smokers. With regard to enzyme exposure, the enzyme-plus detergent-exposed workers showed significant increase in MDA level and SOD activity and insignificant elevation in activities of CAT and GPX as compared with enzyme-free detergent-exposed workers in the second and third monitoring times. The mean values of plasma selenium, iron, and vitamin C levels did not differ significantly between controls and their counterpart subgroups in detergent-exposed workers and were within normal ranges over this study.

**Table 4:** Biological indicators of antioxidant enzymes, lipid peroxidation, and the levels of vitamin C, iron, and selenium in controls and detergent-exposed workers

Biological	Enzyme (±)			Enzyme (−)			Controls		
			
indicator	First monitoring	Second monitoring	Third monitoring	First monitoring	Second monitoring	Third monitoring	First monitoring	Second monitoring	Third monitoring
	C.S (*n* = 57)	C.S (*n* = 57)	C.S (*n* = 57)	C.S (*n* = 58)	C.S (*n* = 58)	C.S (*n* = 58)	C.S (*n* = 16)	C.S (*n* = 16)	C.S (*n* = 16)
	N.S (*n* = 81)	N.S (*n* = 81)	N.S (*n* = 81)	N.S (*n* = 95)	N.S (*n* = 95)	N.S (*n* = 95)	N.S (*n* = 29)	N.S (*n* = 29)	N.S (*n* = 29)
SOD(U/g.Hb)	754# ±115	1178 ± †181	851 ± †128	772 ± 134	1034 ± †193	830 ± 133	729 ± 103	758 ± 108	778 ± 115
	726 ± 108	982 ± †153	792 ± †120	743 ± 122	932 ± †168	788 ± †116	718 ± 124	689 ± 94	731 ± 105
CAT (k/g.Hb)	143.6 ± 17.8	177.8 ± †22.1	155.8 ± †23.3	140.2 ± 23.4	168.2 ± †21.1	147.6 ± †21.3	133.5 ± 17.8	146.3 ± 24.1	130.7 ± 17.2
	136.4 ± 21.5	159.3 ± †20.8	148.1 ± †21.2	134.2 ± 18.3	151.5 ± †21.8	140.2 ± 22.1	141.3 ± 18.6	138.6 ± 21.6	148.3 ± 20.2
GPX (U/L)	10.9 ± 2.14	13.4 ± †2.48	12.0 ± †2.08	10.3 ± 1.75	12.4 ± †1.85	11.3 ± †2.18	9.6 ± 1.71	8.8 ± 1.42	9.2 ± 1.85
	10.4 ± 2.26	12.6 ± †2.27	11.4 ± †1.92	9.6 ± 1.58	11.8 ± †1.94	10.6 ± †2.07	9.1 ± 1.53	9.7 ± 1.73	8.6 ± 1.58
Selenium (*μ*g/L)	96.4 ± 28.8	92.5 ± 29.7	86.8 ± 28.8	87.4 ± 26.8	104 ± 32.7	97.1 ± 21.6	89.3 ± 28.2	85.2 ± 22.5	96.8 ± 28.1
	88.3 ± 31.2	101.2 ± 32.4	89.6 ± 25.9	89.9 ± 29.2	94.7 ± 30.2	87.4 ± 26.2	95.3 ± 24.1	92.7 ± 25.5	86.4 ± 24.2
V.C(mg/L)	1.22 ± 0.62	0.95 ± 0.52	1.08 ± 0.46	1.15 ± 0.49	1.08 ± 0.45	1.12 ± 0.51	1.16 ± 0.38	1.26 ± 0.28	0.96 ± 0.36
	1.08 ± 0.56	1.16 ± 0.58	1.26 ± 0.53	0.95 ± 0.41	1.28 ± 0.52	1.04 ± 0.57	0.92 ± 0.34	1.07 ± 0.25	1.18 ± 0.42
MDA (*μ*mol/L)	0.89 ± 0.31	1.48 ± †0.40	1.22 ± †0.28	0.84 ± 0.19	1.35 ± †0.37	1.08 ± †0.35	0.82 ± 0.24	0.88 ± 0.31	0.79 ± 0.30
	0.78 ± 0.34	1.33 ± †0.31	1.12 ± †0.25	0.73 ± 0.22	1.20 ± †0.32	0.95 ± 0.30	0.77 ± 0.29	0.75 ± 0.33	0.84 ± 0.34
Plasma iron (*μ*g/dl)	141.2 ± 33.8	128.6 ± 32.6	133.6 ± 31.3	137.2 ± 29.4	140.5 ± 33.2	123.3 ± 27.8	144.2 ± 35.7	130.8 ± 32.2	142.3 ± 33.2
	134.6 ± 28.8	124.7 ± 32.2	126.5 ± 27.8	129.4 ± 28.7	129.6 ± 32.5	138.4 ± 29.5	133.5 ± 29.3	138.2 ± 29.1	127.8 ± 30.6

SOD, erythrocyte superoxide dismutase; CAT, catalase; GPX, glutathione peroxidase; V.C, plasma vitamin C; MDA, plasma malondialdehyde; C.S, current smokers; N.S, non-smokers.

# Values are mean ± SD and a level of *P* <0.05 was considered significant.

† Detergent- and/or enzyme-exposed subgroups with mean values of their parameters differing significantly from values of their counterpart subgroups in controls at each monitoring time.

As displayed in Table 5, the investigated indicators in smokers and non-smokers did not show significant differences in each group of detergent-exposed workers and controls at the first monitoring time. Both subgroups in E-plus and E-free detergent fields exhibited significantly higher mean levels (± SD) of 8OH-2'dG, endonuclease III-sensitive sites, and DNA strand breaks at second monitoring as compared with their counterpart subgroups at first and/or third monitoring times and in comparison with controls. Although there was elevation in all indicators of Table 5 in E-plus with respect to E-free detergent workers, they were only significantly different in the values of four recent indicated parameters at the second and/or third monitoring times. All determined parameters were higher in current smokers as compared with non-smokers in all detergent workers at the second and third monitoring times, among which the differences between two groups were significant for the values of DNA strand breaks, endonuclease III-sensitive sites, and 8OH-2'dG. These three recent parameter levels at the end of the study were significantly different and higher in both detergent-exposed workers with respect to the first monitoring time.

**Table 5:** The levels of cytogenetic parameters in detergent-exposed workers and controls

Parameter	Enzyme-plus detergent-exposed workers						Enzyme-free detergent-exposed workers						Controls					
			
	First monitoring		Second monitoring		Third monitoring		First monitoring		Second monitoring		Third monitoring		First monitoring		Second monitoring		Third monitoring	
	C.S (n=57)	N.S (n=81)	C.S (n=57)	N.S (n=81)	C.S (n=57)	N.S (n=81)	C.S (n=58)	N.S (n=95)	C.S (n=58)	N.S (n=95)	C.S (n=58)	N.S (n=95)	C.S (n=16)	N.S (n=29)	C.S (n=16)	N.S (n=29)	C.S (n=16)	N.S (n=29)
DNA S. B	10.11 ± 2.66	9.28 ± 3.38	14.88 ¶±† 3.83	13.64 ¶±† 2.84	12.44 ¶±† 2.73	10.85 ¶± 2.56	9.52 ± 2.36	8.87 ± 2.15	13.27 ¶±† 3.45	12.17 ¶±† 3.13	10.89 ¶± 2.81	9.92 ¶± 2.70	9.15 ± 2.37	8.74 ± 2.04	9.58 ± 2.68	8.38 ± 2.21	8.66 ± 2.40	9.37 ± 2.35
Endo-III-S	1.38 ± 0.54	1.24 ± 0.48	3.61 ¶±† 1.19	2.82 ¶±† 0.94	2.46 ¶± 0.70	1.86 ¶± 0.56	1.23 ± 0.50	1.10 ± 0.42	3.04 ¶±† 0.89	2.30 ¶±† 0.66	2.20 ¶± 0.56	1.66 ¶± 0.52	1.46 ± 0.52	1.34 ± 0.48	1.54 ± 0.58	1.22 ± 0.46	1.71 ± 0.64	1.34 ± 0.56
FPG-S	0.61 ± 0.23	0.50 ± 0.18	0.70 ± 0.29	0.62 ± 0.23	0.63 ± 0.24	0.58 ± 0.19	0.56 ± 0.20	0.62 ± 0.27	0.65 ± 0.28	0.58 ± 0.20	0.61 ± 0.23	0.66 ± 0.29	0.59 ± 0.23	0.66 ± 0.26	0.62 ± 0.24	0.56 ± 0.22	0.67 ± 0.26	0.60 ± 0.23
8OH-2'dG	4.19 ± 0.71	3.97 ± 0.58	7.87 ¶±† 0.73	7.23 ¶±† 0.67	5.43 ¶± 0.53	5.11 ¶± 0.55	4.46 ± 0.48	4.17 ± 0.43	6.68 ¶±† 0.64	6.15 ¶±† 0.57	5.14 ¶± 0.50	4.81 ¶± 0.47	4.28 ± 0.34	4.08 ± 0.39	4.63 ± 0.48	4.37 ± 0.37	4.10 ± 0.38	3.87 ± 0.46

DNA S.B, DNA strand breaks (%DNA in tail); Endo. III-S, endonuclease III-sensitive sites (%DNA in tail); FPG-S, formamidopyrimidine glycosylase-sensitive sites (%DNA in tail); 8OH-2'dG, 8-hydroxy-2'-deoxyguanosine (ng/mg. Cratinine); C.S, current smokers; N.S, nonsmokers.;

#Values are mean ± SD and a level of *P*<0.05 was considered significant.;

† Detergent and/or enzyme-exposed subgroups with mean values of their parameters differing significantly from the values of their counterpart subgroups at first and/or third monitoring time and from controls.;

¶ E-plus detergent-exposed subgroups with mean values of their parameters differing significantly from values of their counterpart subgroups in E-free detergent workers at second and/or third monitoring.

The results of relation analysis between two biomarkers of oxidative stress: MDA, SOD, DNA damage biomarker, and 8OH-2'dG with each other and with CEI are summarized in Table 6 separately. All the analysis showed positive correlation in each monitoring time. The correlation values of the relation between the two indicated biological indicators of oxidative stress and/or between each of them with 8OH-2'dG increased from the first to the second and also to the third monitoring time. The correlation values of 8OH-2'dG-CEI in the field of E-free detergent and/or E-plus detergent field were lower in the second monitoring in comparison with the first monitoring and then increased from the second to the third monitoring.

**Table 6:** Relationship between biological indicators and cumulative exposure index in different monitoring times in both detergent-exposed workers

Correlation	R2					
	
	First monitoring		Second monitoring		Third monitoring	
			
	E-plus	E-free	E-plus	E-free	E-plus	E-free
SOD–MDA	0.22	0.25	0.32	0.30	0.39	0.35
SOD–(8OH-2'dG)	0.27	0.21	0.37	0.31	0.41	0.35
MDA–(8OH-2'dG)	0.20	0.24	0.36	0.32	0.39	0.36
SOD–RD. CEI	0.35	0.33	0.19	0.15	0.28	0.26
(8OH-2'dG)–RD. CEI	0.34	0.28	0.22	0.17	0.28	0.25	
(8OH-2'dG)–RD. CEI	0.29	-	0.16	-	0.20	-

MDA, malondialdehyde; SOD, superoxide dismutase; CAT, catalase; RE, respiratory enzyme; RD, respiratory detergent; 8OH-2'dG 8-hydroxy-2'-deoxyguanosine.

## DISCUSSION

In this study, we selected workers from two separate plants of detergent production, enzyme plus and enzyme-free. They were exposed to detergent dusts of the work place such that the mean levels in each form of respirable and non-respirable dusts were significantly and considerably greater than TLV announced by the ACGIH[26] for about 7.2 years before bag filter installation in their workplace. In addition, with regard to detergent absorption from drinking water, detergent analysis of water revealed that the levels of this compound were under detection limit. Accordingly, the individuals of detergent-exposed workers could not obtain detergent from drinking water. On this basis, lung is the main entry route for detergent and enzyme containing particles and the main site for ROS generation by accumulated and activated inflammatory cells.[35] These ROS may be involved in lipid peroxidation[36] and DNA damage and cause development in antioxidant enzymes.[37] Our follow-up of antioxidant enzyme activities and MDA level confirmed that all parameters increased significantly in both detergent-exposed workers at the second monitoring with a stimulating effect of enzyme exposure and smoking. It is therefore possible that exposure to airborne respiratory particles could be associated with increased antioxidant enzyme activities to convert ROS into inactive metabolites. Enzyme dusts may exert a potentiating effect on ROS generation via their protein properties, which increase the inflammatory reactions.[38] In addition, the lipid-like environment provided by detergents can stabilize antioxidant enzymes, leading to an elevation of their intrinsic activity.[39] On the other hand, the stimulatory effects of smoking may be attributed to the increased deposition of respiratory particles,[40] elevation in activated blood polymorph nuclear cells that release ROS,[41] and direct ROS production by composition of cigarette tobacco.[42] We also noted the possible antioxidant roles of selenium[43] and vitamin C.[44] Because of insignificant variations in plasma Se and vitamin C, they could not contribute to blood antioxidant variations. Neither could metal ions in drinking water that were in normal levels.

There is increasing evidence that ROS are involved in DNA damage by alterations of bases and strand breakage. DNA base adduct resulting from hydroxyl radical damage can be checked by evaluation of 8OH-2'dG[45] and strand breakage by tests such as comet assay.[46]

Our investigation showed that both groups of detergent workers revealed considerable increase in all evaluated DNA damage parameters, with an enhancement effect of enzyme dusts to detergent exposure. Among them, the increase in the levels of 8OH-2'dG, endonuclease III-sensitive sites, and DNA-breaks at second monitoring were significant with respect to the first and/or third monitoring and controls. These observations were in agreement with the studies of Camus *et al*.[47] and Loeckie *et al*.[48] and indicated that phagocyte hyperactivation in the respiratory system contributes to increased ROS generation, which permeate into cell nuclei, induce oxidative DNA damage, and increase urinary excretion of 8OH-2'dG. However, increase in the urinary level of 8OH-2'dG is usually associated with exposure to oxidative conditions.[48] Accordingly, we observed positive relation analysis values between 8OH-2'dG level with SOD activity and MDA levels, respectively, over this study. The stimulatory effect of smoking on DNA damage was in accordance with the study of Maura *et al*.,[49] which indicated the induction of oxidative DNA damage biomarkers such as 8OH-2'dG content in healthy men smokers.

By incorporating FPG and endonuclease III in the analysis of DNA migration,[50] our study further identified considerable oxidative base damage. Although DNA strand breaks showed significant increase in E-plus with respect to E-free detergent-exposed workers, oxidized pyrimidines, as determined by endonuclease III-sensitive sites, seemed to account for most of the oxidative DNA damage induced by detergent and enzyme exposure. It is possible that ROS produced in response to work place pollutants are more likely to cause oxidation of pyrimidines than purines. However, this notion contradicts the general belief that purines such as guanines are more easily oxidized because 8OH-2'dG is the most abundant oxidative modified DNA lesion.[51] Alternatively, the less marked increase in FPG-sensitive sites, coupled with a prominent post race increase in the urinary excretion of 8OH-2'dG, the most abundant repair product of oxidative damage to purines, seemed to imply that oxidized purines were repaired more effectively than oxidized pyrimidines. Our findings were consistent with the recent report of Kelvin *et al*.,[52] demonstrating that the changes in the levels of endonuclease III-sensitive sites in peripheral leukocytes were more pronounced after massive aerobic exercise than FPG-sensitive sites and believed that oxidized purines were repaired more effectively than oxidized pyrimidines. The significant effect of smoking on two parameters of comet assay in our investigation were in accordance with its firmly established genotoxic effect, which also contributed to the DNA damage induced by tobacco smoke exposure.[53]

Following this dramatic overexposure to work place dusts for many years, bag-filter system installation in both plants of the work place was able to reach the level of total dust and their values of incorporated enzyme below SDIA and TLV levels, respectively. In the new work place condition, the reduction in antioxidant enzyme activities and MDA was accompanied with the decrease in CEI and decline in internal DNA damage parameters. On this basis, it is our conviction that these reductions were in response to improvement effects of the filtration system. The findings of the SDIA also showed a pronounced reduction in enzyme- and detergent-related sensitization corresponding to the environment improvement in the United Kingdom detergent factories.[54]

As suggestions, we underline medical surveillance program, periodic medical examinations, entrapment of inhaled particles with high efficiency to protect workers from excessive DNA damage, and, highly recommended, to stop smoking. It is helpful to consider meals enriched with vitamins E and C as ROS scavengers and inactivating factors. Additional studies on separate enzyme exposure from detergents are needed to understand individual genotoxic contributions and mechanisms.

## CONCLUSIONS

Workers exposed to high levels of enzyme-free and to enzyme-plus detergent dusts in two separate plants for several years showed significantly higher SOD, CAT, and GPX activities and lipid peroxidation in comparison with controls and with counterpart groups at the first monitoring. At the second monitoring, enzyme-plus detergent workers had significantly higher levels of these antioxidant enzyme activities and MDA as compared with enzyme-free detergent workers. In this monitoring time, the two groups were also different with controls and with first monitoring time in the levels of investigated DNA damage parameters.

With regard to our results, the observed increase in ROS generation and lipid peroxidation in this study can be considered as the main reason for initiation and promotion of DNA damage in any form. It is our conviction that detergent and enzyme contents of the workplace had a cumulative effect and that smoking had a synergistic effect on oxidative generation and initiation and progression of DNA damage in studied workers. We also propose that the observed decrease in the DNA damage parameters, particularly after filter installation, seems to be related to the filter system ability to reduce airborne detergent and enzyme dusts and the consequence of obvious decrease in the antioxidant enzyme activities and MDA levels (as indicators of decreasing free radicals generation) at the end of this study. In agreement to our propose, it has been demonstrated that inactivation or decreasing of free radicals can also modulate and improve the levels of DNA damage parameters after exposure to oxidative agents.[55] However, the overall data suggest that detergent production processes may contribute to an increase in the total burden of genotoxic damage among workers.
